# Differential diagnosis and prognosis of delayed neuropsychiatric sequelae after acute carbon monoxide poisoning in a patient with schizophrenia: A case report

**DOI:** 10.1002/pcn5.218

**Published:** 2024-06-22

**Authors:** Yuto Satake, Yoshimasa Mamiya, Shizuka Kano, Katsuhiko Akizuki, Mamoru Hashimoto, Manabu Ikeda

**Affiliations:** ^1^ Department of Psychiatry Osaka University Graduate School of Medicine Suita Japan; ^2^ Department of Emergency Ishikiriseiki Hospital Higashiosaka Japan; ^3^ Present address: Department of Child and Adolescent Psychiatry Osaka City General Hospital Osaka Japan; ^4^ Present address: Department of Neuropsychiatry Kindai University Faculty of Medicine Osakasayama Japan

**Keywords:** carbon monoxide poisoning, delayed neuropsychiatric sequelae, electroencephalography, hyperbaric oxygen therapy, schizophrenia

## Abstract

**Background:**

Delayed neuropsychiatric sequelae (DNS) is a syndrome that appears days to weeks after acute carbon monoxide (CO) poisoning. DNS shows various neuropsychiatric symptoms, such as mental deterioration and parkinsonism.

**Case Presentation:**

Our case was a 37‐year‐old male with schizophrenia. He attempted suicide by CO poisoning and was brought to our emergency department (Day 0). He was ventilated with normobaric oxygen therapy for 3 days and moved to the psychiatric ward with clear consciousness. We restarted antipsychotics, and he gradually presented akinesia and rigidity. Additionally, around Day 32, he showed disorganized behaviors, mental deterioration, incontinence, and gait disturbance. Brain magnetic resonance imaging (MRI) showed slightly abnormal findings on Day 35. Although we suspected DNS on the clinical course and the MRI findings, catatonia and side‐effects of antipsychotics were also considered. Finally, electroencephalography (EEG) on Day 38 with apparent abnormalities, including diffuse slow waves, resulted in our diagnosis of DNS, and he underwent hyperbaric oxygen therapy. His condition was dramatically improved, and his diffuse slow waves on EEG disappeared on Day 83. We also followed his clinical presentations and brain MRI until 33 months. Throughout the whole follow‐up, his cognition, movement, and psychiatric symptoms remained stable. However, his brain MRI showed progressive atrophy in bilateral frontal lobes and increasing white matter lesions throughout the whole course.

**Conclusion:**

EEG, as well as brain MRI, may be crucial in the differential diagnosis of DNS in patients with complex conditions involving medications and severe mental illnesses.

## Graphical abstract

The study presents a case of delayed neuropsychiatric sequelae (DNS) following carbon monoxide poisoning in a 37‐year‐old male with schizophrenia. Despite initial uncertainty in diagnosis, electroencephalography (EEG) and magnetic resonance imaging (MRI) abnormalities led to a DNS diagnosis, followed by successful hyperbaric oxygen therapy. The study highlights the importance of EEG and MRI in the differential diagnosis of DNS, especially in patients with complex medical and psychiatric conditions.

## BACKGROUND

Some patients with acute carbon monoxide (CO) poisoning develop delayed neuropsychiatric sequelae (DNS) 2 days to 6 weeks after CO exposure, which manifests as a variety of neuropsychiatric symptoms, including mental deterioration, cognitive impairment, urinary and fecal incontinence, gait disturbance, personality changes, and parkinsonism.^1^ There are no defined diagnostic criteria for DNS, and it can be diagnosed with the characteristic findings in the abovementioned period. Herein, we report a patient with schizophrenia attempting charcoal burning whose DNS was difficult to differentiate from catatonia and drug‐induced parkinsonism.

## CASE PRESENTATION

**Figure 1:**
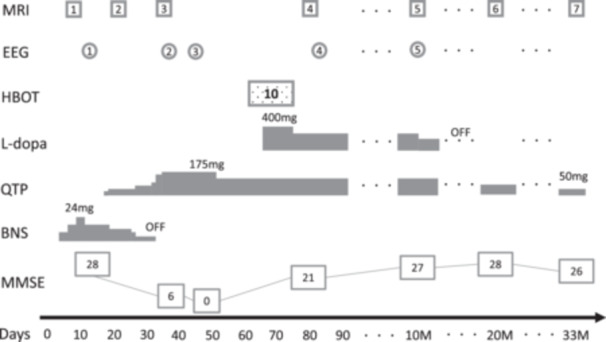
Clinical course. BNS, blonanserin; EEG, electroencephalography; HBOT, hyperbaric oxygen therapy; MMSE, Mini‐Mental State Examination; MRI, magnetic resonance imaging; QTP, quetiapine.

The patient was a 37‐year‐old male with no remarkable physical history. At age 26, persecutory auditory hallucinations with his ex‐girlfriend's voice had started. From the age of 28, he had not been able to work and had started staying at home with his parents. Although his attending psychiatrist had diagnosed him with panic disorder because he had not complained of auditory hallucinations, at age 37, he began to express his auditory hallucinations and a sense of observation, and antipsychotics were added. However, 7 months later (Day 0), he attempted suicide with charcoal burning at home and was brought to the Department of Emergency, Osaka University Hospital. He was diagnosed with CO poisoning with a loss of consciousness and 16.2% CO‐Hb on arrival. He was ventilated to be well‐oxygenated, and consciousness was recovered. His prescription from the clinic was 24 mg of blonanserin, 2 mg of brexpiprazole, 100 mg of amoxapine, 2 mg of ethyl loflazepate, 5 mg of nitrazepam, and 2 mg of lormetazepam. The ventilator was removed on Day 3, and he showed clear consciousness but complained of auditory hallucinations, such as “Kill yourself” by various voices. Although his previous psychiatrist did not mention the possibility of psychotic disorders in the consultation letter, we diagnosed him with schizophrenia based on his clinical course at this point. The doctor seemed to have prescribed antipsychotics for his psychosis as a symptomatic treatment and had not changed the diagnosis due to his expression of the psychotic symptoms for a shorter period compared to his whole disease course. He had never mentioned those symptoms to his parents. We restarted only blonanserin with a lower dose than before admission. Brain magnetic resonance imaging (MRI) showed high‐intensity lesions at bilateral globus pallidus on diffusion‐weighted imaging (DWI) on Day 9. He moved to our psychiatric ward on Day 12. His clinical course after admission is shown in Figure 1.

**Figure 2:**
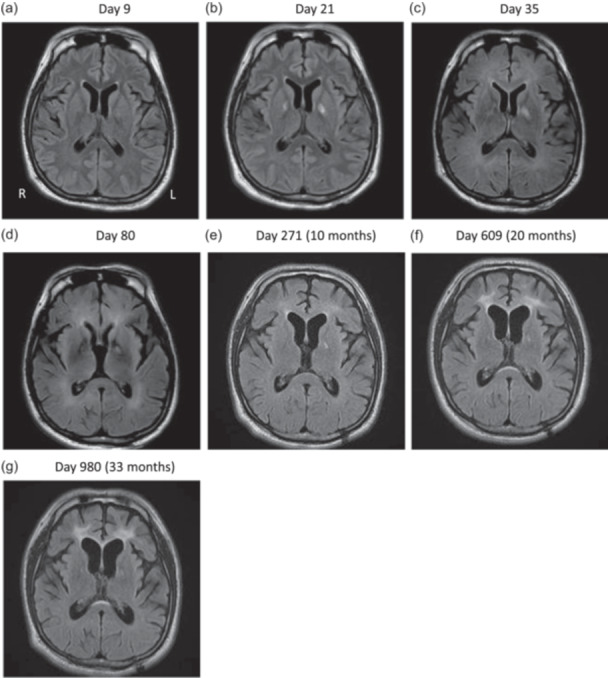
T2‐weighted fluid‐attenuated inversion recovery (T2 FLAIR) magnetic resonance (MR) images. Images (a) to (d) and (e) to (g) were taken with different MR scanners. Legions in bilateral globus pallidus were detectable at Day 9, most prominent at Day 21 and less in later images. Anterior horn was widening during the whole course supported by Evans index (0.276 at Day9, 0.294 at 10 months, 0.319 at 20 months, and 0.332 at 33 months). High intensities in periventricular and centrum semiovale white matter appeared on Day 35, and thicker periventricular white matter lesions in frontal lobes were found at 20 and 33 months.

**Figure 3:**
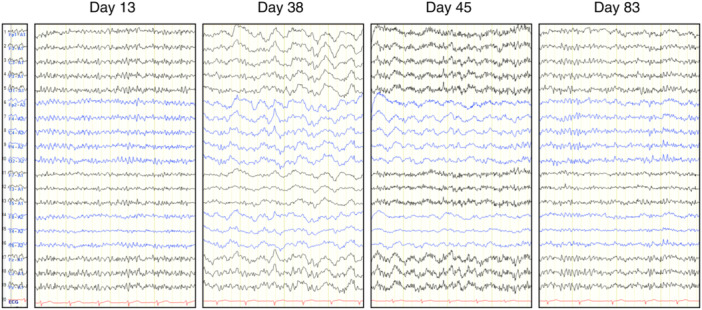
Electroencephalography. All of them are deviated unipolarly referring to electrode at earlobes. On Day 13, basic rhythm was 11–12 Hz with no noticeable slow waves; on Days 38 and 45, basic rhythms were 9–10 Hz with frequent θ and δ waves; on Day 83, basic rhythm was 10–11 Hz with most slow waves disappearing.

He showed akinesia, rigidity, and psychomotor retardation besides hallucinations, and we substituted blonanserin for quetiapine, suspecting drug‐induced parkinsonism. Electroencephalography (EEG) was almost normal with a basic rhythm on Day 13. Mini‐Mental State Examination (MMSE) score was 28 on Day 14. Brain MRI showed high‐intensity lesions even on T2‐weighted and T2‐weighted fluid‐attenuated inversion recovery (FLAIR) images on Day 21. At this point, we started suspecting DNS because of this imaging change. Although his akinesia and rigidity responded to the reduction of blonanserin at first, he started to show disorganized repetitive behaviors, such as putting chopsticks into his underwear, repetitively touching a screw on his walker, and urinating in a wash basin on Day 32. Mutism, gait disturbance, and urine incontinence also appeared on those days. At this point, we also suspected catatonia due to his mutism, stereotypy, and muscle tone resistance, like waxy flexibility. MRI showed a slight high intensity that appeared in diffuse deep white matter on T2 FLAIR images on Day 35. It was difficult to judge whether his deteriorating neuropsychiatric symptoms were from DNS, catatonia, drug‐induced parkinsonism, or compromised factors even at this point. On the contrary, EEG dramatically changed to slower with a basic rhythm of 9–10 Hz and frequent θ and δ range slow waves on Day 38. His MMSE score was 6 on Day 39. He rapidly became inactive and finally had aspiration pneumonia. MDS Unified Parkinsonism Disease Rating Scale (UPDRS) Part III was 75 and did not respond to an intravenous injection of 100 mg of levodopa on Day 41. EEG showed slow waves on Day 45 again, and his MMSE score on Day 49 was zero. The appearance of slow waves on EEG supported organic factors regarding this mental deterioration, and we judged drug‐induced parkinsonism and catatonia were not the main factors explaining his symptoms. High‐signal areas in white matter in T2 FLAIR MR image suggested DNS. Finally, we concluded his series of symptoms after CO poisoning, such as psychomotor retardation, parkinsonism, disorganized behaviors; mutism, were due to DNS. Brain MRI and EEG changes are shown in Figures 2 and 3.

On Day 59, he moved to another general hospital and took 10 sessions of 90‐min hyperbaric oxygen therapy (HBOT) at 2.0 atmospheres absolute by Day 75. His consciousness gradually improved by the seventh session and had rapidly improved by the 10th session. Four hundred milligrams of levodopa was started during this period as symptomatic treatment, with unclear response. He came back to Osaka University Hospital on Day 77. He scored 21 on MMSE, and his hallucinations scarcely annoyed him at that point. MRI on Day 80 showed thicker diffuse high intensity in deep white matter on T2 FLAIR image, and EEG showed basic rhythm fasting up to 10–11 Hz and the disappearance of most of the slow waves on Day 83. We continued 125 mg of quetiapine and moved him to a rehabilitation hospital on Day 91. He finished the physical rehabilitation and was discharged 5 months after the transfer with the ability to walk 3 km by himself. He continued to visit the outpatient clinic without additional HBOT, and we reduced antipsychotics and stopped levodopa during the 2‐year follow‐up period. The final dose of quetiapine was just 50 mg/day, which was enough to maintain the remission, namely, the state without apparent auditory hallucinations and other psychotic symptoms. He did some house chores to help his mother and enjoyed hobbies like watching movies or anime. His parkinsonism showed a good prognosis, and MDS‐UPDRS Part III was 4 at the 10‐months and 0 at the 20‐months assessment. Neuropsychological and imaging follow‐up tests were conducted at 10, 20, and 33 months. Neuropsychological tests were stable throughout the period (Table 1). However, brain MRI showed the progress of brain atrophy and hyperintensities increasing during the whole course (Figure 2). The EEG at 10 months was also similar to that on Day 83.

**Table 1:** Cognitive tests.

Time	Day 14	Day 28	Day 39	Day 49	Day 77	10 months	20 months	33 months
MMSE	28		6	0	21	27	28	26
FAB		4				16	18	17
ACE‐III		80				91	84	81
Attention		17				18	13	14
Memory		17				21	19	16
Fluency		4				10	12	11
Language		26				26	25	25
Visuospatial		16				16	15	15
WAIS‐IV								
Full IQ						86	91	94
VCI						94	96	92
PRI						87	89	93
WMI						79	79	91
PSI						93	108	108

Abbreviations: ACE, Addenbrook's Cognitive Examination; FAB, Frontal Assessment Battery; MMSE, Mini‐Mental State Examination; PRI, Perceptual Reasoning Index; PSI, Processing Speed Index; VCI, Verbal Comprehension Index; WAIS, Wechsler Adult Intelligence Scale; WMI, Working Memory Index.

## DISCUSSION

The various symptoms shown by DNS, such as stupor, parkinsonism‐like muscle tone abnormality, mutism, negativism, and stereotypy, may also derive from catatonia. Also, the possibility of drug‐induced parkinsonism due to the administration of antipsychotic drugs could not be ruled out. However, catatonia and drug‐induced parkinsonism do not show noticeable abnormal changes in brain imaging or EEG. Therefore, in the present case, using these objective tests, we could determine early on that DNS was a primary condition.

Many studies have reported on imaging findings of DNS, and interest in early findings predicting DNS has increased, especially in recent years. High‐intensity areas in globus pallidus, hippocampus, and white matter seen on DWI of acute MRI have been reported to have a sensitivity of 75.2% and specificity of 90.2% for the probability of developing DNS later.^2^ Many patients with DNS also show high‐signal areas in the periventricular and semiventricular white matter.^3^ It sometimes extends to the corpus callosum, subcortical U‐fibers and putamen, some of which are reversible. This is consistent with the hypothesis that an immunological response to the myelin basic protein is one of the mechanisms of DNS.^4^ In our case, the DWI image on Day 9 showed lesions in bilateral globus pallidus and the T2 FLAIR image on Day 35 showed high‐intensity areas in bilateral deep white matter. However, imaging abnormalities are not required to diagnose DNS, and we were not confident in our judgment of DNS as the imaging findings on Day 35 were not very prominent.

With regard to EEG, relatively old articles focused on its diagnostic value in DNS. Konishi et al. reported two cases with normal EEG a week after CO exposure, with diffuse θ waves during a lucid period following DNS.^5^ Min reported in 1986 that 58% of patients with DNS after acute CO poisoning showed abnormal EEG findings, all of which were dominated by generalized δ waves.^6^ This finding suggests subcortical encephalopathy^7^ and has been reported in many other case reports on DNS, including our report. Chang et al. also reported that EEG abnormalities in DNS can improve after HBOT.^8^ EEG basic rhythm changes in 2 weeks may predict the prognosis,^9^ and changes in quantitative EEG alpha frequency after repeated HBOT could indicate efficacy in acute CO poisoning.^10^ In the present case, 5 weeks after the exposure to CO, MRI abnormalities were slight, but EEG abnormalities were evident, and we were convinced of the diagnosis of DNS by the EEG findings. Repeated EEGs, including during the period of clear consciousness, as in the present case, may help to capture the appearance of DNS and the effect of treatment quickly.

## CONCLUSION

We have reported a patient with schizophrenia who attempted a charcoal‐burning suicide and presented with DNS after acute CO poisoning. In psychiatric cases with acute CO poisoning, it can be challenging to differentiate DNS from the aggravation of other disorders and drug‐related problems. MRI and EEG may help make this judgment.

## AUTHOR CONTRIBUTIONS

Yuto Satake examined the patient during the entire observation period and wrote the first manuscript; Yoshimasa Mamiya was the attending physician during the entire observation period. Shizuka Kano was in charge of treatment during hospitalization at Osaka University Hospital and Katsuhiko Akizuki was in charge of HBOT; Mamoru Hashimoto provided substantial input into DNS clinical decisions and treatment planning; Yoshimasa Mamiya and Manabu Ikeda corrected the first draft. All authors approved the final manuscript.

## CONFLICT OF INTEREST STATEMENT

Mamoru Hashimoto is an Editorial Board member of *Psychiatry and Clinical Neurosciences Reports* and a co‐author of this article. To minimize bias, they were excluded from all editorial decision‐making related to the acceptance of this article for publication.

## ETHICS APPROVAL STATEMENT

The Osaka University Clinical Research Review Committee approved this report.

## PATIENT CONSENT STATEMENT

The patient and his family gave written consent to publish the report.

## CLINICAL TRIAL REGISTRATION

N/A

## Data Availability

The original contributions presented in the study are included in the article; further inquiries can be directed to the corresponding author.
